# A new scoring system for predicting short‐term outcomes in Chinese patients with critically‐ill acute decompensated heart failure

**DOI:** 10.1186/s12872-021-02041-2

**Published:** 2021-05-04

**Authors:** Ran Mo, Li-tian Yu, Hui-qiong Tan, Yang Wang, Yan-min Yang, Yan Liang, Jun Zhu

**Affiliations:** Emergency and Intensive Care Center, State Key Laboratory of Cardiovascular Disease, Fuwai Hospital, National Center for Cardiovascular Diseases, Chinese Academy of Medical Sciences and Peking Union Medical College, No. 167 Beilishi Road, Xicheng District, Beijing, People’s Republic of China

**Keywords:** Acute decompensated heart failure, Scoring system, Prognosis

## Abstract

**Background:**

Acute decompensated heart failure (ADHF) contributes millions of emergency department (ED) visits and it is associated with high in-hospital mortality. The aim of this study was to develop and validate a multiparametric score for critically-ill ADHF patients.

**Methods:**

In this single-center, retrospective study, a total of 1268 ADHF patients in China were enrolled and divided into derivation (n = 1014) and validation (n = 254) cohorts. The primary endpoint was any in-hospital death, cardiac arrest or utilization of mechanical support devices. Logistic regression model was preformed to identify risk factors and build the new scoring system. The assigning point of each parameter was determined according to its β coefficient. The discrimination was validated internally using C statistic and calibration was evaluated by the Hosmer-Lemeshow goodness-of-fit test.

**Results:**

We constructed a predictive score based on six significant risk factors [systolic blood pressure (SBP), white blood cell (WBC) count, hematocrit (HCT), total bilirubin (TBIL), estimated glomerular filtration rate (eGFR) and NT-proBNP]. This new model was computed as (1 × SBP < 90 mmHg) + (2 × WBC > 9.2 × 10^9^/L) + (1 × HCT ≤ 0.407) + (2 × TBIL > 34.2 μmol/L) + (2 × eGFR < 15 ml/min/1.73 m^2^) + (1 × NTproBNP ≥ 10728.9 ng/ml). The C statistic for the new score was 0.758 (95% CI 0.667–0.838) higher than APACHE II, AHEAD and ADHERE score. It also demonstrated good calibration for detecting high-risk patients in the validation cohort (χ^2^ = 6.681, p = 0.463).

**Conclusions:**

The new score including SBP, WBC, HCT, TBIL, eGFR and NT-proBNP might be used to predict short-term prognosis of Chinese critically-ill ADHF patients.

**Supplementary Information:**

The online version contains supplementary material available at 10.1186/s12872-021-02041-2.

## Background

Heart failure (HF) is an advanced manifestation of various cardiovascular diseases responsible for several million hospitalizations worldwide, imposing a heavy economic burden [1]. Presence of HF generally implies poor prognosis, especially in patients who are admitted for acute decompensated heart failure (ADHF). Recent data show that the in-hospital mortality for ADHF is 3% and rehospitalization rate exceeds 50% within 6 months [2–4]. According to the latest American College of Cardiology (ACC) guidelines, it is important for the initial evaluation of the clinical trajectory of ADHF. The identification of a high-risk status at admission may help to allocate limited hospital resources and discuss the appropriate goals of care [5]. Therefore, accurately and timely assessing the severity and risk can be beneficial for ADHF patients [6].

Several risk stratification systems have been published previously. Unfortunately, there are several limitations of them. First, few focused on a contemporary intensive care unit (ICU) population with ADHF. Second, the existing risk assessment tools for inpatients with ADHF are often complex and are uniformly underutilized. Third, with new plasma biomarkers emerging and the wide application of bedside echocardiography [7], existing scoring systems need to be updated in line with reassessing all the risk factors. Finally, a recent study demonstrated that clinical care risk scores established to predict the prognosis in unselected ICU patients performed poorly in CICU with ADHF, emphasizing the urgent need to develop improved tools for risk stratification among critically-ill ADHF patients [8]. The aim of this study was to develop and validate a novel clinical scoring model to predict short-term adverse events in a Chinese population of critically-ill ADHF patients and compare it with the existing systems, such as the Acute Physiology and Chronic Health Evaluation (APACHE) system [9], AHEAD score [10] and ACUTEHA score [11].

## Methods

### Study population

Clinical data were collected from 1268 patients with ADHF who were admitted to ICU from the emergency department at Fuwai hospital between January 2014 and December 2018. All participants met the most recent European guidelines for the diagnosis of acute heart failure [12]. Critically-ill ADHF was defined as exacerbation of chronic HF (CHF) with New York Heart Association (NYHA) III/IV symptoms sufficient to be admitted to intensive care. Exclusion criteria were known diagnosis with malignancy. Cases requiring dialysis treatment were excluded from the study population. Patients with ST-segment elevation myocardial infarction and non ST-segment elevation myocardial infarction were also excluded because TIMI score was established, extensively utilized in these patients and reperfusion treatment itself played an important role on the prognosis. However, patients with comorbid coronary heart disease and CHF who were hospitalized for exacerbation of CHF without indications for reperfusion therapy were also included in this study. All data were retrospectively obtained from Fuwai Hospital electronic medical records. The study was approved by the Ethics Committee of Fuwai Hospital and was conducted in accordance with the Declaration of Helsinki.

### Data collection and endpoints

For each patients, baseline information on ED admission was obtained including demographic data, baseline health status, Glasgow coma scale (GCS), body mass index (BMI), vital signs and comorbidities by reviewing their medical records. The primary diagnosis was regarded as the etiology of ADHF even if several pathologies might exist simultaneously. The definition of cardiogenic shock (CS) was consistent with ICU practical guidance [13]. The presence of atrial fibrillation (AF) and bundle branch block (BBB) were measured with 12-lead electrocardiography and pleural effusion was determined by chest X-ray. Left ventricular ejection fraction (LVEF) and estimated pulmonary arterial systolic pressure (PASP) were assessed by using echocardiography (General Electric, USA). The participant`s worst values of blood laboratory tests during the initial 24-h after emergency admission were recorded including arterial pH, PaO_2_, actual bicarbonate (AB), lactate concentration, serum sodium, serum potassium, white blood cell (WBC) count, hemoglobin (Hb) concentration, hematocrit, international normalized ratio (INR), D-dimer concentration, total bilirubin, serum creatinine, serum uric acid (SUA), high-sensitivity troponin I (hs-TNI) and N-terminal pro-B-type natriuretic peptide (NT-proBNP). Estimated glomerular filtration rate was calculated using the Chinese version of the MDRD equation [14].

The main outcome of this analysis was a composite endpoint defined as: (1) in-hospital mortality; (2) in-hospital cardiac arrest; (3)utilization of mechanical support devices during ICU stay which included intra-aortic balloon pumps (IABP) and extracorporeal membrane oxygenation (ECMO). However, some patients transferred from other hospitals who already received mechanical circulatory support before ED visiting were not included in the following analysis. We also collected the information about patients who had listed for heart transplantation (HTx).

### Statistical analysis

For patients’ background data, categorical variables were expressed as frequencies (percentages), and continuous variables were expressed as means ± standard deviations or medians with quartiles depending on their normality. Normality was assessed using the Shapiro–Wilk W-test.

Participants were divided into derivation (Jan 2014–April 2018, n = 1014) and validation (May 2018–December 2018, n = 254) cohorts according to the order of admission to ED. The comparison of the baseline data indicated that the distribution of age and occurrence of endpoint agreed well between the two cohorts but the validation cohort had marginally more female patients, more patients with AF and higher NT-proBNP concentration. Some thresholds for categorical variables were adopted as commonly used in clinical treatment including heart rate (HR), respiratory rate (RR), AB and PaO_2_ whereas age, pH and hs-TNI were considered as continuous variables. Participants were divided into different groups based on the optimal cut-off values of lactate level, serum sodium, WBC, HCT, TBIL, SUA, D-dimer and INR which were determined by respectively performing receiver-operating characteristic (ROC) curve analyses. Patients were defined as underweight by BMI < 18.5 kg/m^2^, normal by 18.5/kg/m^2^ ≤ BMI < 24 kg/m^2^, overweight by BMI ≥ 24 kg/m^2^ and obese by BMI ≥ 30 kg/m^2^. Serum potassium < 3.5mmol/L was defined as hypokalemia and potassium > 5.5 mmol/L was defined as hyperkalemia. The cut-off levels for anemia were hemoglobin < 130 g/L in men and < 120 g/L in women, whereas that for NT-proBNP were determined by quartiles. PASP > 30mmHg was recorded as increased pulmonary artery pressure. The thresholds for eGFR were in accordance with Kidney Outcomes Quality Initiative guidelines, which classified participants into five stages (eGFR ≥ 90, 60 ≤ eGFR < 90, 30 ≤ eGFR < 60, 15 ≤ eGFR < 30 and eGFR < 15 ml/min/1.73 m^2^). Three subgroups based on LVEF were identified: HF with reduced ejection fraction (HFrEF, LVEF < 40%), HF with middle-range ejection fraction (HFmrEF, LVEF 40–49%) and HF with preserved ejection fraction (HFpEF, LVEF ≥ 50%). The predictive power of patients’ characteristics for the short-term adverse outcomes was computed using the univariate logistic regression and described by odds ratios (ORs) and their 95% confidence intervals. Then, the statistically significant predictors identified by univariate analysis were entered into the multivariate logistic regression model with a forward stepwise selection algorithm. Using a method of β-coefficient-based weights similar to that used for the Framingham risk score [15], the assigning weight of each predictor was determined according to the β coefficient in the multivariate logistic regression model to develop a novel scoring system. Subsequently, in order to test the prognostic power of the new score, the ROC methodology was adopted both in derivation and validation groups. The discriminative capacity of the new score was quantified with C-statistic while calibration was graphically evaluated by the Hosmer–Lemeshow goodness-of-fit test.

The software package SPSS version 25.0 (IBM Corporation, New York, NY, USA) was utilized for statistical analysis. All statistical tests were 2-tailed, with a p value < 0.05 considered statistically significant. Graphs were generated using the software GraphPad Prism 8.0.

## Results

### Baseline characteristics

The baseline characteristic of derivation and validation cohorts with critically-ill ADHF were summarized in Table 1. For both groups, the gender, age distribution and risk of adverse outcomes were comparable without significant difference. Of the 1268 patients enrolled, 873 were male with a median age of 58 (± 17) years, among whom the elderly accounted for 17.9%. The top three causes of Chinese ADHF patients were cardiomyopathy (34.5%), ischemic heart disease (30.4%) and valvular disease (17%). CS occurred in 89 patients on admission and 49% were at NYHA IV class on admission. The proportion of HFrEF was 62.3%, 13.9% for HFmrEF and 23.8% for HFpEF. Coexisting atrial fibrillation was observed in 35.6% patients and pleural effusion was identified in 31.9% of the participants.

**Table 1:** Baseline characteristics and incidence of endpoint events

Variable	Value	Derivation cohort (n = 1014)	Validation cohort (n = 254)
Gender (men)	873 (68.8%)	709 (69.9%)	164 (64.6%)
Age (years)	58 ± 17	58 ± 17	58 ± 17
Age ≥ 75 years	227 (17.9%)	179 (17.7%)	48 (18.9%)
*Etiologies (n, %)*			
Ischemic heart disease	385 (30.4)	336 (33.1)	49 (19.3)
Valvular disease	215 (17.0)	161 (15.9)	54 (21.3)
Cardiomyopathy	438 (34.5)	330 (32.5)	108 (42.6)
Arrhythmias	42 (3.3)	39 (3.8)	3 (1.2)
Myocarditis and pericardial disease	29 (2.3)	23 (2.3)	6 (2.5)
Congenital heart disease	62 (4.9)	43 (4.2)	19 (7.4)
Aortic disease	7 (0.6)	6 (0.6)	1 (0.4)
Pulmonary heart disease	33 (2.6)	30 (3.0)	3 (1.2)
Infiltration and toxic damage	56 (4.4)	46 (4.5)	10 (4.1)
Cardiogenic shock (n,%)	89 (7.0)	62 (6.1)	27 (10.6)
NYHA IV (n,%)	621 (49.0)	466 (46.0)	155 (61.0)
BMI (kg/m^2^)	23.90 ± 4.50	24.05 ± 4.50	23.56 ± 4.86
Glasgow coma scale	14.8 ± 0.85	14.8 ± 0.86	14.7 ± 0.86
Temperature (℃)	36.35 ± 0.42	36.33 ± 0.43	36.40 ± 0.38
SBP (mmHg)	115.06 ± 20.46	115.86 ± 20.37	111.88 ± 20.49
HR (BPM)	80 ± 19	80 ± 19	81 ± 21
RR (min^−1^)	19 ± 3	19 ± 3	19 ± 3
Diabetes mellitus (%)	342 (27.0)	273 (26.9)	69 (27.2)
Smoking (%)	632 (49.8)	512 (50.5)	120 (47.2)
Alcohol use (%)	509 (40.1)	412 (40.6)	97 (38.2)
Arterial pH	7.45 ± 0.11	7.44 ± 0.11	7.46 ± 0.05
PaO_2_ (mmHg)	87.46 ± 26.79	88.01 ± 25.38	85.30 ± 31.65
AB (mmol/L)	24.41 ± 4.40	24.44 ± 4.43	24.30 ± 4.33
Lactic acid (mmol/L)	1.82 ± 1.15	1.83 ± 1.20	1.78 ± 1.03
Serum sodium (mmol/L)	136.06 ± 5.05	136.35 ± 5.08	134.92 ± 4.76
Serum potassium (mmol/L)	4.05 ± 0.58	4.06 ± 0.58	4.02 ± 0.60
Leukocyte count (× 10^9^/L)	7.8 ± 3.5	7.8 ± 3.5	8.0 ± 3.7
Hemoglobin (g/L)	134.9 ± 24.7	135.8 ± 24.3	131.5 ± 25.8
Hematocrit	0.409 ± 0.071	0.411 ± 0.070	0.398 ± 0.074
INR	1.39 ± 0.89	1.37 ± 0.96	1.46 ± 0.58
D-dimer (mg/L)	2.24 ± 3.49	2.21 ± 3.83	2.35 ± 2.92
Total bilirubin (μmol/L)	29.5 ± 25.3	29.7 ± 24.0	28.5 ± 29.9
Uric acid (μmol/L)	540.4 ± 196.3	532.1 ± 191.0	573.4 ± 213.3
Hs-TNI (μg/L)	0.045 (0.02–0.10)	0.04 (0.02–0.10)	0.06 (0.02–0.13)
NT-proBNP (ng/ml)	5478.2 (2286.5–11,957.3)	4966.5 (2171.3–10,728.9)	6821.2 (3028.5–17,569.3)
*LVEF*			
HFrEF (yes, %)	790 (62.3)	629 (62.0)	161 (63.4)
HFmrEF (yes, %)	176 (13.9)	141 (13.9)	35 (13.8)
HFpEF (yes, %)	302 (23.8)	244 (24.1)	58 (22.8)
PASP > 30 mmHg (%)	590 (46.5)	456 (45.0)	134 (52.8)
*eGFR (ml/min/1.73 m*^*2*^*)*			
eGFR ≥ 90 (%)	271 (21.4)	232 (22.9)	39 (15.3)
60 ≤ eGFR < 90 (%)	375 (29.6)	283 (27.9)	92 (36.2)
30 ≤ eGFR < 60 (%)	489 (38.6)	383 (37.8)	106 (41.7)
15 ≤ eGFR < 30 (%)	109 (8.6)	97 (9.6)	12 (4.7)
eGFR < 15 (%)	24 (1.9)	19 (1.8)	5 (2.0)
Atrial fibrillation (%)	451 (35.6)	349 (34.5)	102 (40.2)
Pleural effusion (%)	404 (31.9)	322 (31.8)	82 (32.3)
Hospitalization time (days)	13 (9–18)	13 (8–17)	15 (10–20)
*Outcomes*			
Composite endpoint (%)	181 (14.3)	144 (14.2)	37 (14.6)
In-hospital death (%)	117 (9.2)	93 (9.2)	24 (9.4)
In-hospital cardiac arrest (%)	48 (3.8)	41 (4.0)	7 (2.8)
Applications of IABP or ECMO (%)	16 (1.3)	10 (1.0)	6 (2.4)
Heart transplantation (%)	45 (3.5)	32 (3.2)	13 (5.1)

BMI, body mass index; SBP, systolic blood pressure; HR, heart rate; RR, respiratory rate; AB, actual bicarbonate; hs-TNI, high-sensitivity troponin I; NT-proBNP, N-terminal pro-B-type natriuretic peptide; LVEF, left ventricular ejection fraction; PASP, pulmonary arterial systolic pressure; IABP, intra-aortic balloon pump; ECMO, extracorporeal membrane oxygenation

During hospitalization, the primary endpoint occurred in 181 patients (14.3%) with 117 death (9.2%). The heart transplantation occurred in 3.5% of the patients. The median total hospitalization time was 13 (9–18) days.

### Logistic regression and model establishment

Univariate analysis was performed in derivation cohort using the univariate logistic regression model and included the following 30 clinical parameters: age, elderly, sex, BMI, GCS, temperature, SBP, heart rate, RR, arterial pH, PaO_2_, AB, lactic acid, serum sodium, potassium, WBC, Hb, HCT, TBIL, SUA, eGFR, D-dimer, INR, NT-proBNP, hs-TNI, LVEF, PASP, existence of AF, pleural effusion and BBB. All variates except age, elderly, sex, temperature, RR, arterial pH, PaO_2_, hs-TNI, LVEF, AF and BBB were found to be significantly associated with the incidence of short-term adverse outcomes.

Based on the results of univariate analysis, a forward stepwise method was adopted for 19 indexes that showed significant relations for predicting short-term outcomes. Low SBP, high WBC level, HCT, concentrations of TBIL, NT-proBNP and coexistence of stage five chronic kidney disease (CKD) were identified as the independent predictors. Using these six risk factors and with consideration of the weighing of respective β coefficients, we determined assigned points for each parameter, which led to a new prognostic stratification system. Because the weight associated with HCT was the lowest, we specified low HCT to 1 point and divided all weights by a factor of 1.07 then rounding them to the nearest integer. The novel scoring system was as follows:

$$(1 \times {\hbox{SBP}} < 90\,{\hbox{mmHg}})+(2\times {\hbox{WBC}} > 9.2 \times10^{9}/{\hbox{L}})+(1\times {\hbox{HCT}} \leq 0.407)+(2\times {\hbox{TBIL}} > 34.2\,\upmu{\hbox{mol/L}})+(2\times {\hbox{stage 5 CKD}})+(1\times {\hbox{NTproBNP}} \ge10728.9\,{\hbox{ng/ml}}).$$

The univariate and multivariate logistic analysis results were listed in Table 2.

**Table 2:** Odds ratios of covariates for primary endpoint in derivation cohort and model definitions (n = 1014)

Variable	Univariate		Multivariate		Model selection	
	OR (95% CI)	p	OR (95% CI)	p	β value	Score
Female gender	0.838 (0.576–1.221)	0.358				
Age	0.993 (0.983–1.003)	0.183				
Age ≥ 75 years	0.923 (0.576–1.478)	0.738				
*BMI (kg/m*^*2*^*)*						
Normal	0	1				
Underweight	1.713 (0.970–3.024)	0.064				
Overweight	0.810 (0.509–1.289)	0.375				
Obese	0.651 (0.357–1.188)	0.162				
GCS	1.875 (1.490–2.357)	< 0.001				
Temperature	0	0.999				
*SBP*						
SBP < 90 mmHg	5.927 (2.75–12.762)	< 0.001	4.636 (1.881–11.426)	0.001	1.534	1
SBP ≥ 140 mmHg	1.403 (0.765–2.575)	0.274	0.943 (0.352–2.530)	0.908		
*HR (bpm)*						
70 ≤ HR < 110	0	1				
HR < 55	1.17 (0.574–2.386)	0.666				
55 ≤ HR < 70	0.848 (0.541–1.33)	0.473				
110 ≤ HR < 159	2.142 (1.228–3.736)	0.007				
*RR (min*^*−1*^*)*						
24 < RR ≤ 34	1.385 (0.631–3.041)	0.461				
RR > 34		1				
pH	0.322 (0.078–1.333)	0.118				
*PaO*_*2*_						
60 < PaO_2_ ≤ 70	0.580 (0.213–1.581)	0.287				
55 < PaO_2_ ≤ 60	1.181 (0.41–3.404)	0.758				
PaO_2_ ≤ 55	0.706 (0.191–2.614)	0.602				
*AB*						
AB < 22 mmol/l	2.017 (1.295–3.142)	0.002				
AB > 28 mmol/l	1.975 (1.193–3.272)	0.008				
Lactic acid > 2.1 mmol/l	3.047 (1.904–4.876)	< 0.001				
Serum sodium < 136.1 mmol/l	2.530 (1.742–3.674)	< 0.001				
*Serum potassium*						
Hypokalemia	0.797 (0.497–1.277)	0.345				
Hyperkalemia	4.55 (1.513–13.682)	0.007				
WBC > 9.2 × 10^9^/L	6.036 (4.151–8.776)	< 0.001	6.742 (3.719–12.223)	< 0.001	1.908	2
Anemia	1.512 (1.052–2.174)	0.025				
HCT ≤ 40.7%	1.96 (1.365–2.814)	< 0.001	2.915 (1.598–5.319)	< 0.001	1.070	1
TBIL > 34.2 μmol/l	3.037 (2.111–4.370)	< 0.001	5.929 (3.225–10.899)	< 0.001	1.780	2
SUA > 547.9 μmol/l	2.662 (1.850–3.830)	< 0.001				
D-dimer > 0.92 mg/l	4.018 (2.651–6.090)	< 0.001				
INR > 1.145	3.354 (2.195–5.124)	< 0.001				
hs-TNI	1.041 (0.988–1.096)	0.136				
*LVEF*						
HFpEF	0	1				
HFmrEF	0.548 (0.281–1.068)	0.077				
HFrEF	0.936 (0.621–1.411)	0.752				
*PASP*						
PASP ≤ 30 mmHg	0	1				
30 < PASP ≤ 50 mmHg	1.078 (0.671–1.732)	0.757				
50 < PASP ≤ 70 mmHg	0.965 (0.602–1.547)	0.883				
PASP > 70 mmHg	2.638 (1.381–5.039)	0.003				
Pleural effusion	2.016 (1.408–2.887)	< 0.001				
AF	0.830 (0.567–1.216)	0.34				
BBB	0.756 (0.480–1.190)	0.227				
*eGFR (ml/min/1.73 m*^*2*^*)*						
eGFR ≥ 90	0	1	0	1		
60 ≤ eGFR < 90	0.940 (0.502–1.760)	0.846	0.516 (0.217–1.226)	0.134		
30 ≤ eGFR < 60	1.745 (1.014–3.003)	0.044	1.205 (0.555–2.615)	0.638		
15 ≤ eGFR < 30	2.412 (1.210–4.808)	0.012	0.773 (0.251–2.378)	0.653		
eGFR < 15	6.395 (2.23–18.334)	0.001	5.374 (1.116–25.881)	0.036	1.681	2
*NT-proBNP (ng/ml)*						
NT-proBNP < 2171.3	0	1	0	1		
2171.3 ≤ NT-proBNP < 4966.5	1.593 (0.724–3.505)	0.247	1.517 (0.583–3.949)	0.393		
4966.5 ≤ NT-proBNP < 10,728.9	2.018 (0.943–4.320)	0.071	0.867 (0.324–2.320)	0.777		
NT-proBNP ≥ 10,728.9	6.049 (3.03–12.073)	< 0.001	2.920 (1.188–7.179)	0.02	1.072	1

### Discrimination and calibration of the new score

**Fig. 1:**
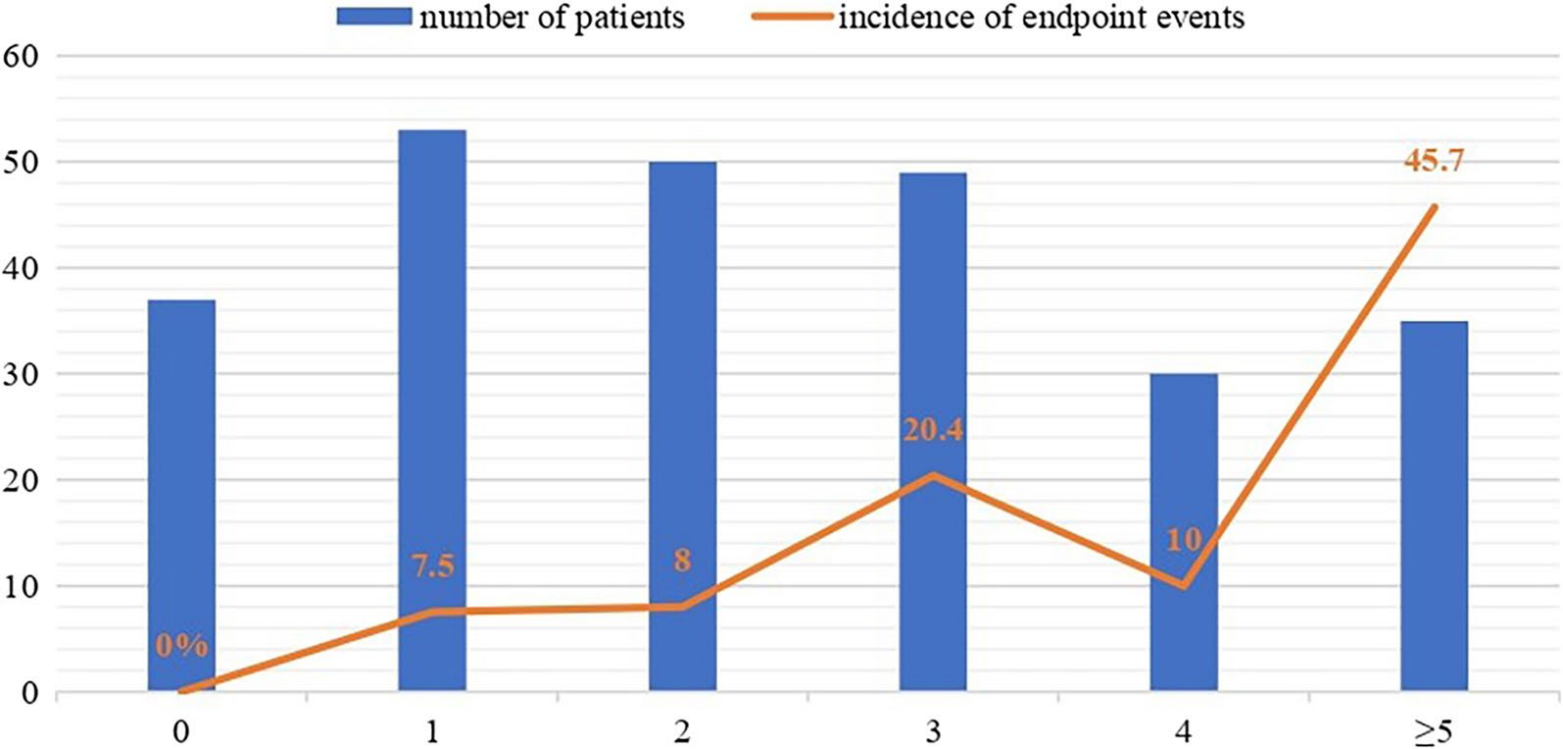
Prevalence of the different scores and incidence of adverse events. Blue bars represent the number of patients for each score. The orange line represents the incidence rates according to the new score

In the derivation cohort, the C statistic of new scoring system was 0.794 (95% CI 0.753–0.836, p < 0.001). Among the validation patients, since cases with scores of 5 or higher were limited, we combined them into one group for subsequent analysis. The incidence of adverse outcomes increased from 0% for score of 0 to 7.5%, 8%, 20.4%, 10 and 45.7% for score of 1, 2, 3, 4, and 5 points or higher. The scores of the validation cohort and the incidence of primary endpoint events were shown in Fig. 1. Additional clinical baseline data of each score was presented in the Additional file 1.

**Fig. 2:**
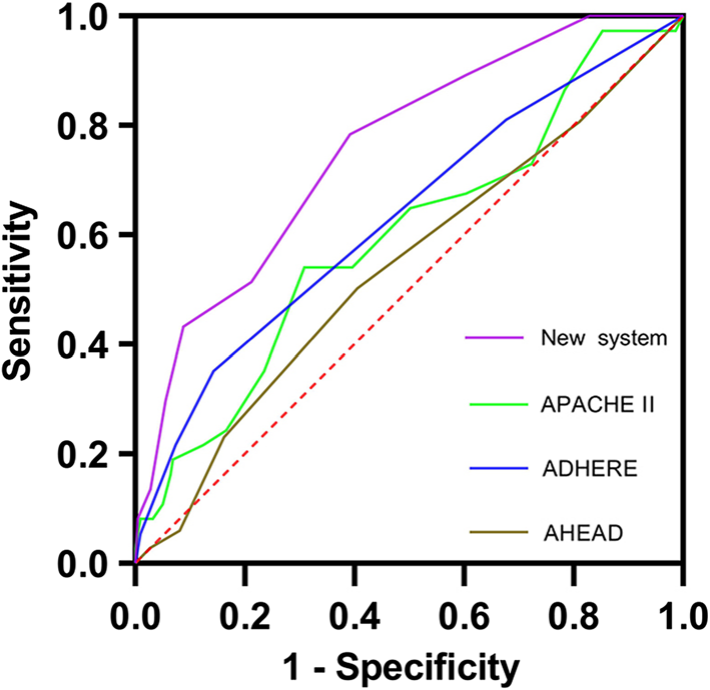
Receiver operating characteristic curves for different scoring systems. The *green line* represents APACHE II. The *blue line* represents ADHERE risk tree. The brown line represents AHEAD score. The purple line presents the new scoring system

**Fig. 3:**
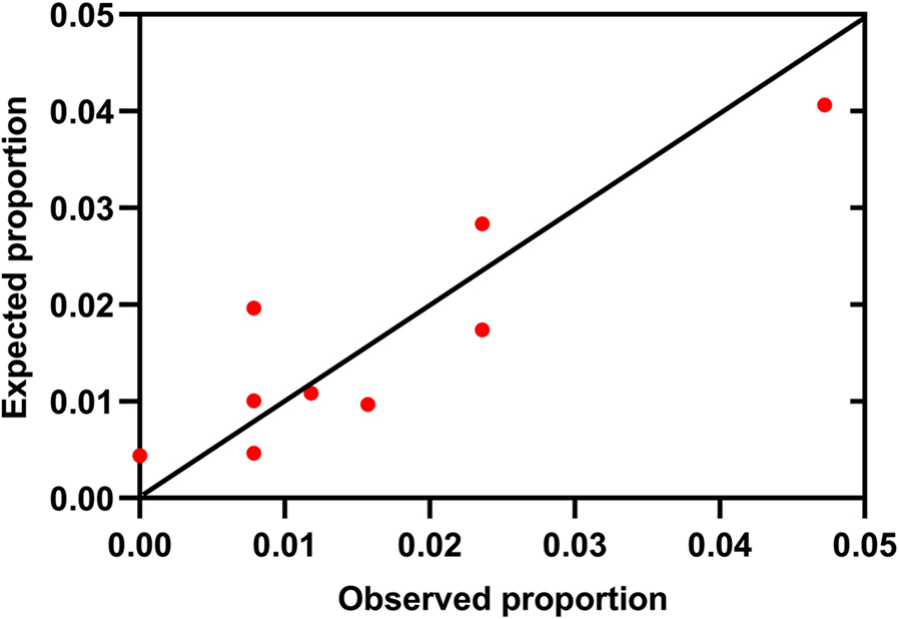
Calibration plots showing the agreement between predicted and observed probabilities for primary endpoints

Using receiver operating characteristics analysis, the C statistics were calculated for comparison of the discriminative power between the new score and other established systems. The C statistic for our new score was 0.758 (95% CI 0.677–0.838, p < 0.001), whereas for APACHE II was 0.598 (95% CI 0.496–0.700, p = 0.058), for ADEHER risk tree [4] and AHEAD score was 0.631 (95% CI 0.529–0.733, p = 0.011) and 0.540 (95% CI 0.442–0.638, p = 0.439) respectively, demonstrating that our system had a better predictive power for short-term outcomes in critically-ill ADHF patients. The comparison of these four scores were shown in Fig. 2. The calibration of the system was evaluated with the Hosmer–Lemeshow goodness-of-fit test. In the validation cohort, the new scoring system demonstrated a good calibration (χ^2^ = 6.681, p = 0.463) for detecting high-risk ADHF patients admitted to ED. The calibration plots were shown in Fig. 3. Mantel–Haenszel test and Pearson correlation test showed a significantly positive relationship between the score and endpoints (in Additional file 1: Table S2).

Furthermore, we attempted to predict the occurrence of heart transplantation with the new system. The C statistics for our system, APACHE II, AHEAD and ADHERE were 0.428 (95% CI 0.319–0.537, p = 0.382), 0.358 (95% CI 0.234–0.482, p = 0.084), 0.480 (95% CI 0.336–0.624, p = 0.809) and 0.501 (95% CI 0.382–0.621, p = 0.986). None of them showing enough ability for predicting the considerations for HTx selection.

## Discussion

In the present study of Chinese patients in a single cardiovascular center ICU setting, we developed and validated a predictive model based on physical examinations and laboratory testing withing 24 h after ED admission. We found that six parameters were significantly associated with poor short-term outcomes: low systolic blood pressure (SBP < 90 mmHg); increasing white blood cell (WBC > 9.2 × 10^9^/L); low hematocrit (HCT ≤ 0.407); abnormal liver function (TBIL > 34.2 µmol/L); NT-proBNP ≥ 10728.9 ng/ml and stage 5 CKD (eGFR < 15 ml/min/1.73 m^2^). In comparison, several commonly used existing tools did not exhibit an adequate ability to predict in-hospital outcomes. The new risk score might aid in the identification of ADHF patients at risk for the incidence of in-hospital death, cardiac arrest or use of mechanical support devices in China.

### The predictive elements for ADHF

Previous studies have shown that multiple related risk factors can effectively predict adverse outcomes of AHF. The clinical importance of SBP has been considered and prognostic scores such as ADHERE [2], AHFI [16] and GWTG-HF [17] have been created. Gheorghiade et al. reported that a systolic pressure under 120 mmHg at the time of admission was associated with a poor prognosis compared with a systolic pressure over 120 mmHg [18]. In addition, renal dysfunction is a well-known strong prognostic parameter of ADHF. A retrospective study with 104,794 AHF patients demonstrated that abnormal eGFR on admission was proved to be a significant predictor of mortality and readmission risk [19]. In keeping with the fact that nearly all established systems took renal function into account [7], we used eGFR instead of serum creatinine as the indicator scoring 2 points if less than 15 ml/min/1.73  m^2^ in both sex. According to China heart failure (China-HF) registry, elevated total bilirubin is an independent predictor of adjusted in-hospital mortality [20]. Samsky et al. also reported that increase of total bilirubin was closely related to 30-day, 180-day mortality and HF rehospitalization. Elevated WBC count is the most common abnormality in AHF. Previous studies showed that WBC reflected the systemic inflammatory response [22, 23], sympathetic overactivity [24] and a physiological reaction to metabolic acidosis [25]. In the present study, it also emerged as one of he most important determinants of short-term prognosis. Anemia is a frequent co-morbidity in AHF patients. Existing evidence has suggested that development of anemia was correlated with increased mortality and higher hospitalization rates irrespectively of age, gender or NYHA functional class in AHF [26]. Although most previous studies used hemoglobin concentrations as an indicator for anemia, our study employed HCT because of its better prognostic performance in ADHF. Little studies clarified the difference between Hb and HCT in AHF settings, but we speculated volume overload or hemodilution might cause the better performance than Hb [27]. Plasma NT-proBNP is another well-known strong predictor of ADHF, and a meta-analysis of ADHF patients has confirmed that NT-proBNP is an independent predictor of mortality both in all-cause and cardiovascular death despite different cut points, time intervals and prognostic models [28]. Although current studies employed different cut-off values for NT-proBNP, we used quartiles for multivariate analysis, which showed that only patients with the highest plasma concentration of NT-proBNP was related to poorer in-hospital outcomes scoring 1 point in the new system.

In addition to the six independent risk factors, there are some clinical indicators that have been attached great importance in clinical practice or included in other scores. Recently, Zymliński et al. reported that, in a study of 237 AHF without overt evidence of peripheral hypoperfusion, blood lactate on admission was associated with markers of organ dysfunction and a worse prognosis [25]. They also found lactic acid was a comprehensive index which was affected by HR, WBC, liver function and big endothelin-1. It might explain the reason why lactate was not an independent risk factor when taking multiple parameters into account. As for LVEF, with the deepening understanding of HFpEF, HFpEF patients showed similar or even worse prognosis compared with HFrEF [29]. In another study with 343 AHF, Uriel et al. found that LVEF was not correlated with outcomes, suggesting cautious interpretation when applying LVEF to evaluate AHF patients [30].

### The unique potential value of the new score

To improve the prognosis for ADHF, it is crucial to identify high-risk patients as a first step. Several risk stratification systems have been published for AHF previously such as the Acute Physiology and Chronic Health Evaluation (APACHE) system [9], AHEAD score [10], ADHERE, American Heart Association Get With the Guidelines-Heart Failure (GWTG-HF) [17]. ACUTE HF score [11] and AHFI [16]. In our study, we chose APACHE II, ADHERE and AHEAD as comparisons and discovered better predictive capability of our score in the Chinese ADHF patients. Firstly, the study population in our analysis was critically-ill ADHF patients admitted to ICU who had more complex comorbidities and more severe symptoms. Secondly, these three clinical predictive models for AHF were derived and externally validated in North American or European patients, their performance might vary substantially across different world regions. A recent study indicated that region-specific recalibrations were needed for AHF scoring systems [31]. Additionally, APACHE II was published in 1985 while ADHERE in 2005 and AHEAD in 2016. With development and application of multiple new diagnosing techniques and arising plasma biomarkers, some clinical indicators should be brought into reevaluation such as NT-proBNP, hs-TNI and D-dimer.

In view of the urgent and special ED cases, we paid more attention to objective laboratory tests rather than personal past medical history. On the one hand, disease histories are often collected through self-reporting which may cause omission or missing of previous medical history considering the urgent ED clinical cases. On the other hand, Table 1 showed that elderly patients accounted for a large proportion, who might experience memory loss or disturbance of consciousness. These reasons made collecting past medical history accurately and completely a tough task at ED visit. Therefore, we did not include predictive scores containing many medical histories such as GWTG-HF, AHFI and OPTIMIZE-HF into our analysis.

There are several noteworthy features of the present investigation: Because of the exclusion criteria not covering LVEF, it was carried out in a cohort of ADHF patients containing not only HFrEF but also HFpEF and HFmrEF often ignored in other studies. And it offered a relatively comprehensive system for evaluating in-hospital outcomes for critically-ill ADHF patients, due to the complete analysis of clinical, biochemical, electrocardiographic and echocardiographic parameters. Considering the incompleteness and availability of past medical history in practical ED situations, we did not highlight past diseases in building the new scoring system. Also, we utilized logistic regression instead of regression tree analysis, hence constructed a quantifiable tool to reach a better predictive accuracy. Moreover, the final model consisting of six easy-to-obtain indexes with a simple calculation method was relatively convenient to identify high-risk populations and aid to determine whether an ADHF admitted to ICU should be closely monitored and managed. For these reasons, our new score might represent a practical and efficient approach to the critically-ill patients commonly hospitalized for ADHF in China.

### New score and heart transplantation

Although this new system showed a satisfactory predictive power for the composite endpoint, it cannot accurately predict HTx. The candidacy for HTx was assessed carefully in Fuwai hospital. Elderly and frail patients with ADHF who failed optimal medical management and mechanical circulatory support often suffered from malnutrition, immune dysfunction and multiple organ failure. They were obviously unsuitable for operations. It was understandable that the score was unparallel to the consideration of HTx. Secondly, the selection for HTx was not only associated with HF conditions but also economic conditions, social support and psychological condition (Additional file 1: Table S3).

### Study limitations

This study presents a potential model for triaging emergency department ADHF patients for intensive care unit. Also, it had several limitations. First, our database consisted of a cohort of patients from a single cardiovascular hospital, and the study population included only Chinese patients. The participants evaluated was limited to patients admitted only to the ICU, and ADHF patients who were then admitted to other wards were not enrolled. Although an internal validation was performed by bootstrapping techniques in the same population, the results should be carefully interpreted when applied to external validation studies. Second, the composite endpoint of our study was in-hospital death or cardiac arrest or clinical application of mechanical support devices. The selected 6 parameters demonstrated good ability to distinguish patients with high risk of short-term adverse events. Due to lack of follow-ups after discharge, the ability of our scoring system to predict post-discharge and long-term prognosis was still uncertain. Third, the individual clinical data was collected at the time of admission without counting the effects of pre-hospital managements, such as the widely used inotropic drugs for ADHF, which may influence admission blood pressure and heart rate. Besides, there were still some potentially significant clinical parameters we did not collect such as systemic inflammation as measured by CRP or PCT, frailty index and consciousness score. Further studies will be needed to evaluate these factors for the next scoring system.

## Conclusions

Existing predictive systems did not demonstrate enough ability to evaluate the incidences of short-term adverse events in critically-ill ADHF in Chinese population. Our new scoring system including SBP, white blood cell count, hematocrit, total bilirubin, estimated glomerular filtration rate and NT-proBNP might provide a practical tool for daily risk stratification of ADHF patients, irrespective of its etiology.

## Supplementary Information

**Additional file 1**: Table S1. Baseline characteristics of each score in the validation group. Table S2. The correlations between the new score and short-term outcomes. Table S3. Baseline characteristics in HTx patients.

## Data Availability

The datasets used and analysed during the current study are available from the corresponding author on reasonable request.

## Abbreviations

ADHF: acute decompensated heart failure
ED: Emergency department
SBP: Systolic blood pressure
HCT: Hematocrit
WBC: White blood cell
TBIL: Total bilirubin
eGFR: Estimated glomerular filtration rate
HF: Heart failure
ACC: American College of Cardiology
ICU: Intensive care unit
APACHE: Acute Physiology and Chronic Health Evaluation
CHF: Chronic heart failure
GCS: Glasgow coma scale
BMI: Body mass index
CS: Cardiogenic shock
AF: Atrial fibrillation;
BBB: Bundle branch block
LVEF: Left ventricular ejection fraction
PASP: Pulmonary arterial systolic pressure
AB: Actual bicarbonate
Hb: Hemoglobin
HCT: Hematocrit
INR: International normalized ratio
SUA: Serum uric acid
hs-TNI: High-sensitivity troponin I
NT-proBNP: N-terminal pro-B-type natriuretic peptide
IABP: Intra-aortic balloon pumps
ECMO: Extracorporeal membrane oxygenation
HTx: Heart transplantation
HR: Heart rate
RR: Respiratory rate
ROC: Receiver-operating characteristic
CKD: Chronic kidney disease
ORs: Odds ratios
CI: Confidence interval
HFrEF: Heart failure with reduced ventricular ejection fraction
HFpEF: Heart failure with preserved ejection fraction
HFmrEF: Heart failure with mid-range ejection fraction
