# Supplementary material for: A new scoring system for predicting short‐term outcomes in Chinese patients with critically‐ill acute decompensated heart failure
Source: BMC Cardiovasc Disord. 2021 May 4;21:228. doi: 10.1186/s12872-021-02041-2 (PMC8094523; doi:10.1186/s12872-021-02041-2)
Supplement: Supplementary file 1 — Additional file 1: Table S1. Baseline characteristics of each score in the validation group. Table S2. The correlations between the new score and short-term outcomes. Table S3. Baseline characteristics in HTx patients. [file 12872_2021_2041_MOESM1_ESM.docx]

**Supplementary Material**

Table 1. Baseline characteristics of each score in the validation group(n=254)

|  | 0(n=37) | 1(n=53) | 2(n=50) | 3(n=49) | 4(n=30) | ≥5(n=35) |
| --- | --- | --- | --- | --- | --- | --- |
| Gender(men,%) | 28(75.7) | 36(67.9) | 35(70) | 34(69.4) | 15(50) | 16(45.7) |
| Age(years) | 55±19 | 60±15 | 55±18 | 56±13 | 66±17 | 59±20 |
| CS(n,%) | 0(0) | 5(9.4) | 6(12) | 8(16.3) | 2(6.7) | 7(17.1) |
| BMI(kg/m^2^) | 25.01±5.16 | 24.49±4.03 | 24.3±4.90 | 21.96±5.57 | 20.51±3.72 | 19.30±7.06 |
| GCS | 15 | 15 | 15 | 15 | 15 | 14 |
| T(℃) | 36.2±0.4 | 36.3±0.2 | 36.4±0.3 | 36.4±0.3 | 36.4±0.3 | 36.7±0.5 |
| SBP(mmHg) | 119±20 | 115±22 | 112±22 | 106±19 | 113±18 | 107±19 |
| HR(BPM) | 80±22 | 74±18 | 87±22 | 82±21 | 79±13 | 88±26 |
| RR(min^-1^) | 19±2 | 19±3 | 20±4 | 20±4 | 20±3 | 20±4 |
| DM(%) | 10(27.0) | 20(37.7) | 12(24) | 11(22.4) | 10(33.3) | 5(14.3) |
| Smoking(%) | 19(51.4) | 28(52.8) | 24(48.0) | 25(51.0) | 11(36.7) | 13(37.1) |
| Alcohol use(%) | 18(48.6) | 20(37.7) | 22(44.0) | 22(44.9) | 9(30.0) | 7(20.0) |
| Arterial PH | 7.46±0.04 | 7.46±0.05 | 7.46±0.05 | 7.46±0.06 | 7.46±0.05 | 7.47±0.07 |
| PaO_2_(mmHg) | 86.9±15.8 | 87.8±24.3 | 81.6±25.2 | 87.4±50.9 | 77.7±22.9 | 88.5±34.5 |
| AB(mmol/L) | 25.1±3.7 | 25.1±3.8 | 24.1±4.3 | 23.8±4.3 | 23.8±4.0 | 23.6±5.8 |
| Lactate(mmol/L) | 1.5±0.5 | 1.5±0.7 | 1.8±0.7 | 1.9±1.1 | 1.8±0.9 | 2.3±1.8 |
| Serum sodium(mmol/L) | 137±3 | 135±5 | 135±4 | 134±5 | 134±5 | 134±5 |
| Serum potassium(mmol/L) | 4.1±0.6 | 3.9±0.5 | 3.9±0.6 | 4.1±0.6 | 4.2±0.6 | 4.1±0.8 |
| WBC (×10^9^/L) | 6.79±1.32 | 6.06±1.73 | 8.62±4.68 | 8.48±3.26 | 7.62±4.49 | 10.93±4.08 |
| Hb(g/L) | 149±11 | 123±20 | 140±26 | 135±26 | 112±26 | 126±28 |
| HCT(%) | 0.447±0.029 | 0.374±0.058 | 0.424±0.0.75 | 0.406±0.076 | 0.344±0.077 | 0.378±0.081 |
| INR | 1.22±0.36 | 1.40±0.50 | 1.46±0.67 | 1.46±0.56 | 1.54±0.50 | 1.71±0.76 |
| D-dimer(mg/L) | 0.90±0.96 | 1.46±2.14 | 1.98±1.99 | 2.97±3.05 | 3.03±4.06 | 4.74±3.77 |
| TBIL(μmol/L) | 19.05±6.32 | 16.93±7.65 | 31.09±50.07 | 30.93±19.34 | 25.56±19.15 | 51.89±34.73 |
| SUA(μmol/L) | 535.60±160.20 | 476.31±200.53 | 569.36±163.31 | 589.88±213.17 | 630.36±211.11 | 681.83±280.00 |
| Hs-TNI(μg/L) | 0.184±0.734 | 0.096±0.230 | 0.521±1.957 | 0.148±0.189 | 0.134±0.267 | 0.603±1.424 |
| NT-proBNP(ng/ml) | 3712.6(2126.5-5580.5) | 3600.5(2153.5-6789.0) | 8540.9(4309.1-12723) | 8414.5(4057.2-19179.5) | 18954.5(12471.2-39478.8) | 30970.7(11147.5-45000.0) |
| HFrEF(n,%) | 25(67.6) | 28(52.8) | 36(72.0) | 33(67.3) | 15(50) | 23(65.7) |
| HFmrEF(n,%) | 7(18.9) | 8(15.1) | 4(8.0) | 6(12.2) | 5(16.7) | 4(11.4) |
| HFpEF(n,%) | 5(13.5) | 17(32.1) | 10(20.0) | 10(20.4) | 10(33.3) | 8(22.9) |
| PASP>30mmHg(%) | 16(43.2) | 25(47.2) | 23(46.0) | 27(55.1) | 24(80.0) | 18(51.4) |
| eGFR≥90(%) | 6(16.2) | 9(17.0) | 10(20.0) | 4(8.2) | 1(3.3) | 2(5.7) |
| 60≤eGFR<90 | 18(48.6) | 14(26.4) | 16(32.0) | 19(38.8) | 2(6.7) | 4(11.4) |
| 30≤eGFR<60 | 13(35.1) | 30(56.6) | 24(48.0) | 20(40.8) | 12(40.0) | 6(17.1) |
| 15≤eGFR<300 | 0 | 0 | 0 | 3(6.1) | 14(46.7) | 16(45.7) |
| eGFR<15 | 0 | 0 | 0 | 3(6.1) | 1(3.3) | 7(20.0) |
| AF(%) | 15(40.5) | 25(47.2) | 14(28.0) | 23(46.9) | 12(40.0) | 14(40.0) |
| Pleural effusion(%) | 6(16.2) | 19(35.8) | 16(32.0) | 15(30.6) | 14(46.7) | 13(37.1) |
| Hospitalization days | 15±8 | 19±12 | 18±12 | 16±11 | 14±7 | 15±12 |
| Composite endpoints(%) | 0 | 4(7.5) | 4(8.0) | 10(20.4) | 3(10.0) | 16(45.7) |
| In-hospital death(%) | 0 | 1(1.9) | 3(6.0) | 6(12.2) | 3(10.0) | 11(31.4) |
| In-hospital cardiac arrest(%) | 0 | 1(1.9) | 0 | 3(6.1) | 0 | 3(8.6) |
| Application of IABP or ECMO(%) | 0 | 2(3.8) | 1(2.0) | 1(2.0) | 0 | 2(5.7) |

Table 2. The correlations between the new score and short-term outcomes

|  | χ^2^ | Degrees of freedom | p value |
| --- | --- | --- | --- |
| Mantel-Haenszel test | 27.361 | 1 | <0.001 |
|  | Number of cases | r | p value |
| Pearson correlation | 254 | 0.329 | <0.001 |

Table 3. Baseline characteristics in HTx patients

| Variable | Total population | HTx patients(n=45) |
| --- | --- | --- |
| Gender(men) | 873(68.8%) | 35(77.8%) |
| Age(years) | 58±17 | 50±17 |
| Age≥75years | 227(17.9%) | 1(2.22%) |
| Etiologies(n,%) |  |  |
| Ischemic heart disease | 385(30.4) | 9(20.0) |
| Valvular disease | 215(17.0) | 5(11.1) |
| Cardiomyopathy | 438(34.5) | 28(62.2) |
| Arrhythmias | 42(3.3) | 0 |
| Myocarditis and pericardial disease | 29(2.3) | 0 |
| Congenital heart disease | 62(4.9) | 1(2.2) |
| Aortic disease | 7(0.6) | 0 |
| Pulmonary heart disease | 33(2.6) | 0 |
| Infiltration and toxic damage | 56(4.4) | 2(4.4) |
| Cardiogenic shock(n,%) | 89(7.0) | 6(13.3) |
| BMI(kg/m^2^) | 23.90±4.50 | 22.89±4.52 |
| Glasgow coma scale | 14.8±0.85 | 14.9±0.86 |
| Temperature(℃) | 36.35±0.42 | 36.36±0.42 |
| SBP(mmHg) | 115.06±20.46 | 101.51±20.31 |
| HR (BPM) | 80±19 | 78±19 |
| RR (min^-1^) | 19±3 | 18±3 |
| Diabetes mellitus(%) | 342(27.0) | 11(24.4) |
| Smoking(%) | 632(49.8) | 20(44.4) |
| Alcohol use(%) | 509(40.1) | 19(42.2) |
| Arterial pH | 7.45±0.11 | 7.47±0.11 |
| PaO_2_(mmHg) | 87.46±26.79 | 96.43±24.37 |
| AB(mmol/L) | 24.41±4.40 | 23.10±4.42 |
| Lactic acid(mmol/L) | 1.82±1.15 | 1.97±1.17 |
| Serum sodium(mmol/L) | 136.06±5.05 | 134.53±5.07 |
| Serum potassium(mmol/L) | 4.05±0.58 | 3.95±0.58 |
| Leukocyte count(×10^9^/L) | 7.8±3.5 | 7.9±3.5 |
| Hemoglobin(g/L) | 134.9±24.7 | 143.5±24.4 |
| Hematocrit | 0.409±0.071 | 0.431±0.070 |
| INR | 1.39±0.89 | 1.36±0.89 |
| D-dimer(mg/L) | 2.24±3.49 | 2.87±3.47 |
| Total bilirubin(μmol/L) | 29.5±25.3 | 33.7±25.4 |
| Uric acid(μmol/L) | 540.4±196.3 | 551.0±196.2 |
| Hs-TNI(μg/L) | 0.045(0.02~0.10) | 0.400(0.02~0.10) |
| NT-proBNP(ng/ml) | 5478.2(2286.5~11957.3) | 9432.4(2383~11936) |
| LVEF |  |  |
| HFrEF (yes, %) | 790(62.3) | 43(95.6) |
| HFmrEF(yes, %) | 176(13.9) | 1(2.2) |
| HFpEF(yes, %) | 302(23.8) | 1(2.2) |
| PASP>30mmHg(%) | 590(46.5) | 25(55.6) |
| eGFR (ml/min/1.73m^2^) |  |  |
| eGFR≥90(%) | 271(21.4) | 12(26.7) |
| 60≤eGFR<90(%) | 375(29.6) | 15(33.3) |
| 30≤eGFR<60(%) | 489(38.6) | 18(40.0) |
| 15≤eGFR<30(%) | 109(8.6) | 0 |
| eGFR<15(%) | 24(1.9) | 0 |
| Atrial fibrillation(%) | 451(35.6) | 15(33.3) |
| Pleural effusion(%) | 404(31.9) | 15(33,3) |
| Hospitalization time(days) | 15±12 | 15±27 |
